# MRI With Gadolinium as a Measure of Blood-Labyrinth Barrier Integrity in Patients With Inner Ear Symptoms: A Scoping Review

**DOI:** 10.3389/fneur.2021.662264

**Published:** 2021-05-20

**Authors:** Christopher I. Song, Jacob M. Pogson, Nicholas S. Andresen, Bryan K. Ward

**Affiliations:** ^1^Department of Otolaryngology-Head and Neck Surgery, Johns Hopkins University School of Medicine, Baltimore, MD, United States; ^2^Department of Neurology, Johns Hopkins University School of Medicine, Baltimore, MD, United States; ^3^Department of Neurology, Royal Prince Alfred Hospital, Camperdown, NSW, Australia

**Keywords:** otosclerosis, Ménière's disease, inner ear, blood-labyrinth barrier, gadolinium, MRI

## Abstract

**Objective:** Capillaries within the inner ear form a semi-permeable barrier called the blood-labyrinth barrier that is less permeable than capillary barriers elsewhere within the human body. Dysfunction of the blood-labyrinth barrier has been proposed as a mechanism for several audio-vestibular disorders. There has been interest in using magnetic resonance imaging (MRI) with intravenous gadolinium-based contrast agents (GBCA) as a marker for the integrity of the blood labyrinth barrier in research and clinical settings. This scoping review evaluates the evidence for using intravenous gadolinium-enhanced MRI to assess the permeability of the blood-labyrinth barrier in healthy and diseased ears.

**Methods:** A systematic search was conducted of three databases: PubMed, EMBASE, CINAHL PLUS. Studies were included that used GBCA to study the inner ear and permeability of the blood-labyrinth barrier. Data was collected on MRI protocols used and inner ear enhancement patterns of healthy and diseased ears in both human and animal studies.

**Results:** The search yielded 14 studies in animals and 53 studies in humans. In healthy animal and human inner ears, contrast-enhanced MRI demonstrated gradual increase in inner ear signal intensity over time that was limited to the perilymph. Signal intensity peaked at 100 min in rodents and 4 h in humans. Compared to controls, patients with idiopathic sudden sensorineural hearing loss and otosclerosis had increased signal intensity both before and shortly after GBCA injection. In patients with Ménière's disease and vestibular schwannoma, studies reported increased signal at 4 h, compared to controls. Quality assessment of included studies determined that all the studies lacked sample size justification and many lacked adequate control groups or blinded assessors of MRI.

**Conclusions:** The included studies provided convincing evidence that gadolinium crosses the blood-labyrinth barrier in healthy ears and more rapidly in some diseased ears. The timing of increased signal differs by disease. There was a lack of evidence that these findings indicate general permeability of the blood-labyrinth barrier. Future studies with consistent and rigorous methods are needed to investigate the relationship between gadolinium uptake and assessments of inner ear function and to better determine whether signal enhancement indicates permeability for molecules other than gadolinium.

## Introduction

Living tissues need a steady supply of nutrients to support metabolism and clear waste. Blood vessels lined by endothelial cells transport these metabolic resources, and capillaries are the site at which nutrients and waste are exchanged. Capillaries have different features depending on the needs of nearby tissues, being more porous, for example in the liver, or more restrictive in the retina (1). In some locations, such as the brain, capillaries form junctions that are so impermeable they create a continuous barrier. In the inner ear, a blood-labyrinth barrier was first proposed to explain differential uptake of intravenously injected compounds between the endolymph and perilymph spaces (2).

**Figure 1:**
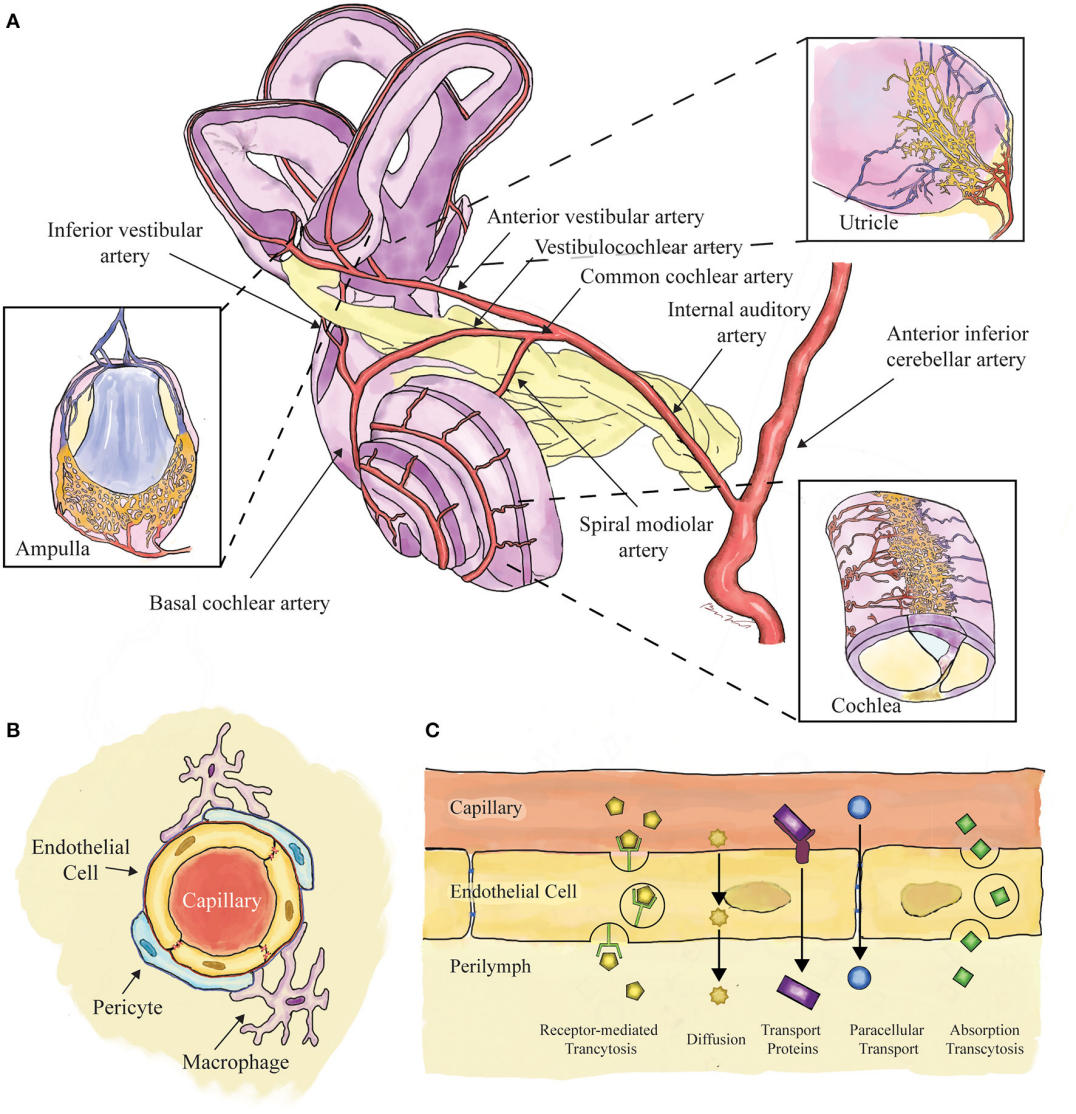
Schematic showing the components of the blood-labyrinth barrier. **(A)** Blood supply to the labyrinth is shown, with insets showing capillary beds near the sensory epithelia of the ampullae, otoconial organs, and cochlea. **(B)** Capillaries of the blood-labyrinth barrier include endothelial cells with tight junctions, surrounded by pericytes and resident macrophages that regulate permeability. **(C)** Examples of hypothetical mechanisms by which molecules can transit across the barrier are shown.

Intravenously injected compounds reach the inner ear via the labyrinthine artery, a branch of the anterior inferior cerebellar artery, that subsequently branches into smaller vessels to supply the labyrinth and cochlea (Figure 1A). Capillary networks in the inner ear are clustered around the stria vascularis and spiral ligament in the cochlea, and the sensory epithelia of the vestibular system (3). These capillary networks are—like in the eye and brain—the presumed location of the blood-labyrinth barrier, composed of endothelial cells with tight-junctions surrounded by pericytes and resident macrophages (4) (Figure 1B). These barriers tightly regulate ion composition within the endolymph and perilymph, and are permeable to water, glucose, and small molecules (5, 6). Molecules transit across the barrier *via* a variety of mechanisms including diffusion, endocytosis, and transcellular protein transport (7) (Figure 1C).

The role of the blood-labyrinth barrier in clinical medicine is receiving increased attention. Studies have shown that several hours following intravenous administration of gadolinium-based contrast agents (GBCA), the perilymphatic space of the inner ear enhances on magnetic resonance imaging (MRI) studies (8). MRI using stronger static magnetic fields and new pulse sequences has improved spatial resolution and takes advantage of this contrast between the endolymph-filled membranous labyrinth and the surrounding perilymphatic space. These MRI techniques have emerged as a useful research tool in the study of Ménière's disease, a disorder in which patients commonly have a swelling of the membranous labyrinth (9). It is presumed that the increased signal intensity reflects GBCA crossing the blood-labyrinth barrier.

More recently, studies have begun exploring the use of GBCA to determine the integrity of the blood-labyrinth barrier in disease. In addition to transmitting small molecules, capillaries also allow the transit of white blood cells into tissues (10). During inflammation, released cytokines can activate capillary endothelial cells, increasing capillary permeability (11). Increased permeability may have deleterious effects on the inner ear (4). An imaging marker of the permeability of the blood-labyrinth barrier could provide diagnostic and prognostic information and aid the development of new therapies for inner ear diseases. The aim of this scoping review was to assess the evidence for the use of GBCA as a marker of permeability of the blood-labyrinth barrier in animals and humans with inner ear disease.

## Methods

A scoping review was performed with the aim of synthesizing knowledge regarding the use of MRI to assess the integrity of the blood-labyrinth barrier. A broad search was performed using three databases: PubMed, EMBASE, and CINAHL Plus. This search was performed on 10/5/2020 using controlled vocabulary (e.g., MeSH terms in PubMed) and keywords related to the concepts of the “inner ear,” “contrast-enhanced MRI,” and the “blood-labyrinth barrier” (see Supplementary Material). The search strategy was created with assistance from staff at the Welch Medical Library at Johns Hopkins Medicine.

Two study members (CS and BW) independently evaluated articles and included those that met the following inclusion criteria: aims to assess the blood-labyrinth barrier with MRI, uses a GBCA administered via intravenous injection, includes original data, is not a case report (i.e., must include data from more than one individual), and is written in English language. Additional articles were included *via* a snowballing approach, where the references in each included article were assessed using the same inclusion criteria.

For both clinical and animal studies we recorded data on enhancement of inner ear structures in diseased and healthy ears, the imaging protocol, MRI static magnetic field strength, the GBCA and dose, and the time from contrast injection to image acquisition.

All co-authors reviewed the included studies and two study members (CS and BW) also evaluated the quality and risk of bias of each clinical research article using a modified version of the National Institutes of Health (NIH) Quality Assessment Tool for Cross-Sectional Studies (12) (Table 1).

**Table 1:** Modifications made to the NIH quality assessment checklist.

**Modified NIH quality assessment checklist**	
**Original question**	**Specific interpretation used**
Was the research question or objective in this paper clearly stated?	Was the assessment of abnormal inner ear enhancement or blood-labyrinth barrier breakdown a specified goal of the research?
Was the study population clearly specified and defined?	Was the study population specified as a single or multi-center sample? Was the sampling method described?
Was the participation rate of eligible persons at least 50%?	
Were all the subjects selected or recruited from the same or similar populations (including the same time period)? Were inclusion and exclusion criteria for being in the study pre-specified and applied uniformly to all participants?	Were the inclusion criteria specific and applied uniformly?
Was a sample size justification, power description, or variance and effect estimates provided?	Was a power analysis included to justify sample sizes?
For the analyses in this paper, were the exposure(s) of interest measured prior to the outcome(s) being measured?	Were patients included in the study prior to MRI assessment?
Was the timeframe sufficient so that one could reasonably expect to see an association between exposure and outcome if it existed?	Did patients undergo MRI within a reasonable time frame from symptom onset in studies of disease states? (i.e., within 30 days for ISSHL)
For exposures that can vary in amount or level, did the study examine different levels of the exposure as related to the outcome (e.g., categories of exposure, or exposure measured as continuous variable)?	Were both pre-contrast and post-contrast MRI evaluated?
Were the exposure measures (independent variables) clearly defined, valid, reliable, and implemented consistently across all study participants?	Were the MRI protocol and contrast dose and agent clearly described?
Was the exposure(s) assessed more than once over time?	Was MRI performed and assessed at varying time points following contrast administration?
Were the outcome measures (dependent variables) clearly defined, valid, reliable, and implemented consistently across all study participants?	Was the measurement of contrast enhancement done with clearly specified and reliable methods?
Were the outcome assessors blinded to the exposure status of participants?	Was it clearly specified that MRI findings were evaluated by individuals blinded to the clinical status of patients?
Was loss to follow-up after baseline 20% or less?	
Were key potential confounding variables measured and adjusted statistically for their impact on the relationship between exposure(s) and outcome(s)?	Were reasonable controls used as comparisons to diseased ears?

*If no interpretations are specified, the original question was sufficient for our purposes. MRI, magnetic resonance imaging, ISSHL, idiopathic sudden sensorineural hearing loss*.

Since the goal of this scoping review was to qualitatively describe the current literature and map key concepts in this field, test statistics were not performed.

## Results

### Literature Search

**Figure 2:**
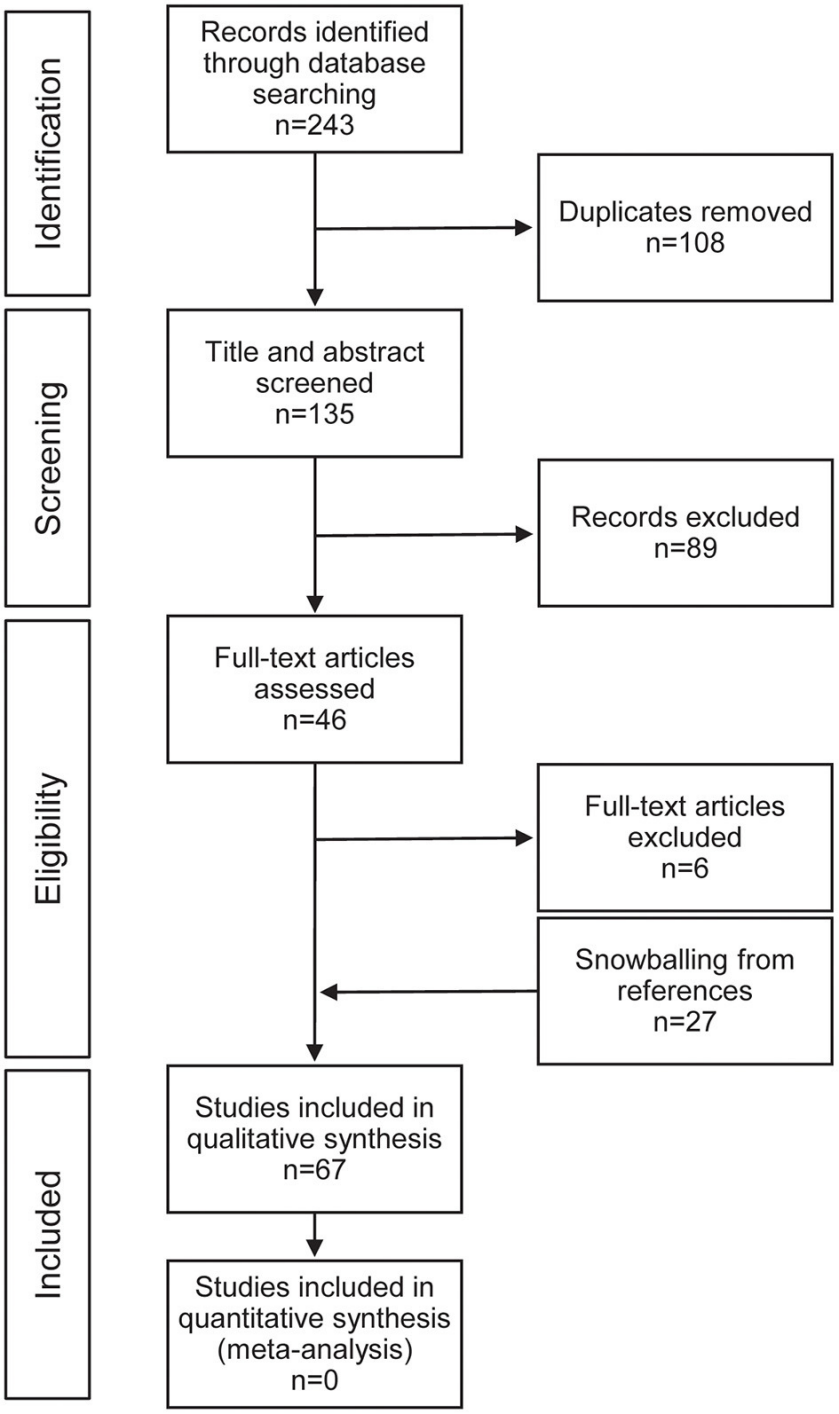
Preferred reporting items for systematic reviews and meta-analyses (PRISMA) flow diagram for study selection.

Our database search yielded 243 citations, 88 from PubMed, 109 from EMBASE, and 46 from CINAHL Plus. After duplicate articles were removed, there were 135 unique search results. After full text review with the application of selection criteria we had 40 included publications. Additional review of the references from the initial included publications yielded an additional 27 citations that met the selection criteria and were included in the final analysis (Figure 2).

### Animal Studies

There were 14 animal studies in this review that evaluated MRI of healthy and diseased inner ears of guinea pigs and/or mice. Eleven of these studies administered contrast agent in doses of 1.5 mmol/kg and used MRI scanners of magnetic field strengths 4.7 Tesla (T) or greater. Studies in healthy ears of guinea pigs and mice reported increased signal intensity within the perilymph, consistent with GBCA uptake, that increased over time and peaked at 100 min, whereas no enhancement was seen in the endolymph (13–18). Enhancement was first seen in the cochlea with relative greater signal in the scala tympani than the scala vestibuli, followed by the utricle and saccule, and the ampullated ends of the semicircular canals (19, 20). The areas correspond to capillary networks in the inner ear, suggesting entry of GBCA at the blood-labyrinth barrier. Comparison of different GBCA found some variation in signal intensity and uptake kinetics, but intravenous administration of both macrocyclic and linear agents reliably led to enhancement of perilymph (20, 21). In mice, all GBCAs led to enhancement of perilymph with increasing intensity over time from 15–105 min (20). Gadobutrol (Gadovist) was shown to have the most rapid uptake and greatest signal enhancement while gadopentetate dimeglumine (Magnevist) had the slowest and lowest intensity of enhancement (20). In three studies, mechanical trauma to and inflammation of the inner ear were found to increase the rate of signal intensity rise in the inner ear following intravenous GBCA over 15–80 min above the rate in control animals (14, 19, 22).

### Human Studies

Our search yielded 53 studies in humans. Of these, 43 were cross-sectional studies and 10 were descriptive case series. Case series were defined as studies that provided only descriptive characteristics of included patients. The case series provided a useful historical framework for subsequent studies but were not included in our analysis (23–32). In healthy human ears, described in two included studies, enhancement of the inner ear peaked at 4 h following intravenous injection of 0.1 and 0.3 mmol/kg GBCA using 3T MRI and 3D fluid-attenuated inversion recovery (FLAIR) pulse sequences (8, 33).

Our quality assessment found flaws in methodological rigor across the included studies (Table 2). Assessment of the included cross-sectional studies with the NIH quality assessment tool found that none (0%) of the studies provided sample size justification, 20 (47%) either did not specify whether the MRI assessment was blinded or had used assessors that were not blinded, and 18 (42%) lacked a control population for comparing abnormal enhancement. Additionally, 35 (81%) did not assess enhancement at multiple time points, and 21 (49%) did not provide data on signal intensity with different levels of contrast exposure (e.g., signal intensity before and after contrast administration).

**Table 2:** Quality assessment of included human studies with the NIH quality assessment tool.

**Author**	**Clear Research Question**	**Clearly Specified Pop**.	**Participation >50%**	**Uniform Inclusion Criteria**	**Sample Size Justification**	**Exposure measured before outcome**	**Sufficient Timeframe**	**Varied Exposure Levels Measured**	**Clearly Defined Exposure**	**Exposure Assessed Over time**	**Clearly Defined Outcome**	**Assessors Blinded**	** <20% Loss to Follow-up**	**Confounders Addressed**
Sartoretti-Schefer (34)	N	N	NA	N	N	Y	Y	Y	N	N	N	N	NA	Y
Sartoretti-Schefer (35)	N	Y	NA	Y	N	Y	N	Y	N	N	N	N	NA	N
Fitzgerald and Mark (36)	N	N	NA	Y	N	Y	N	N	N	N	N	N	NA	N
Stokroos et al. (37)	Y	N	NA	Y	N	Y	N	N	N	N	N	N	NA	N
Strupp et al. (38)	Y	N	NA	Y	N	Y	Y	N	Y	N	N	N	NA	N
Schick et al. (39)	N	N	NA	N	N	Y	N	N	N	N	N	Y	NA	N
Naganawa et al. (40)	Y	Y	NA	Y	N	NA	NA	Y	Y	Y	N	N	NA	NA
Naganawa et al. (33)	Y	Y	NA	Y	N	NA	NA	Y	Y	Y	Y	N	NA	NA
Cadoni et al. (41)	N	Y	NA	Y	N	Y	Y	Y	N	N	N	N	NA	N
Sugiura et al. (42)	Y	Y	NA	Y	N	Y	Y	Y	Y	N	N	N	NA	Y
Sone et al. (43)	Y	Y	NA	N	N	Y	Y	Y	Y	N	N	N	NA	N
Carfrae et al. (8)	Y	N	NA	Y	N	Y	Y	N	Y	N	N	Y	NA	Y
Yoshida et al. (44)	Y	Y	NA	Y	N	Y	Y	Y	Y	Y	Y	N	NA	N
Yamazaki et al. (45)	Y	Y	NA	Y	N	Y	Y	Y	Y	N	Y	N	NA	Y
Lee et al. (46)	Y	Y	NA	N	N	Y	N	Y	Y	N	Y	N	NA	Y
Nakata et al. (47)	N	N	NA	Y	N	Y	Y	Y	Y	N	Y	Y	NA	Y
Tagaya et al. (48)	Y	N	NA	N	N	Y	Y	N	Y	N	Y	Y	NA	Y
Tanigawa et al. (49)	Y	Y	NA	Y	N	Y	N	Y	Y	N	Y	Y	NA	N
Suzuki et al. (50)	Y	Y	NA	N	N	Y	Y	N	Y	N	Y	N	NA	N
Tagaya et al. (51)	Y	N	NA	N	N	Y	Y	N	Y	N	Y	Y	NA	Y
Naganawa et al. (52)	Y	Y	NA	N	N	Y	Y	Y	Y	Y	Y	Y	NA	N
Sano et al. (53)	Y	N	NA	N	N	Y	N	Y	Y	Y	Y	Y	NA	Y
Berrettini et al. (54)	Y	Y	NA	Y	N	Y	Y	Y	Y	N	Y	N	NA	Y
Ishikawa et al. (55)	Y	Y	NA	Y	N	Y	Y	Y	Y	N	Y	N	NA	Y
Kim et al. (56)	Y	N	Y	Y	N	Y	N	Y	Y	Y	Y	Y	NA	Y
Tanigawa et al. (57)	Y	N	NA	Y	N	Y	N	Y	Y	N	Y	Y	NA	N
Liao et al. (58)	N	N	Y	Y	N	Y	Y	Y	Y	N	Y	Y	NA	Y
Lombardo et al. (59)	Y	Y	Y	Y	N	Y	Y	N	Y	N	Y	Y	NA	Y
Naganawa et al. (60)	Y	Y	Y	Y	N	Y	Y	N	Y	N	Y	N	NA	Y
Pakdaman et al. (61)	Y	Y	Y	Y	N	Y	N	N	Y	N	Y	N	NA	Y
Attye et al. (62)	N	Y	NA	Y	N	Y	Y	N	Y	N	N	Y	NA	Y
Berrettini et al. (63)	Y	N	NA	Y	N	Y	Y	N	Y	N	Y	Y	NA	Y
Byun et al. (64)	Y	Y	Y	Y	N	Y	Y	Y	N	Y	N	Y	NA	Y
Eliezer et al. (65)	Y	Y	NA	Y	N	Y	Y	Y	Y	N	Y	Y	NA	Y
Bernaerts et al. (66)	Y	Y	NA	Y	N	Y	Y	N	Y	N	N	Y	NA	N
Conte et al. (67)	Y	Y	Y	Y	N	Y	Y	N	Y	N	Y	N	NA	Y
Eliezer et al. (68)	Y	Y	NA	Y	N	Y	N	N	Y	N	N	Y	NA	Y
Wang et al. (69)	Y	Y	NA	Y	N	Y	N	N	N	N	Y	Y	NA	Y
Bowen et al. (70)	Y	N	NA	Y	N	Y	Y	Y	Y	Y	Y	N	NA	Y
Eliezer et al. (71)	Y	N	NA	Y	N	Y	Y	N	Y	N	N	Y	NA	N
Eliezer et al. (72)	N	N	NA	N	N	N	N	N	Y	N	N	Y	NA	N
Kahn et al. (73)	N	Y	NA	Y	N	Y	Y	N	Y	N	N	Y	NA	N
Laine et al. (74)	Y	Y	NA	Y	N	Y	Y	N	Y	N	Y	Y	NA	Y

*Details of each quality assessment category is explained in Table 1. Studies that did not describe participation and follow-up details were described as “not available” (NA) for “>50% patient participation” and “<20% loss to follow up.” For studies of healthy human ears, “not available” (NA) was used to describe “Exposure measured before outcome” and “Sufficient Timeframe”*.

Contrast-enhanced MRI in patients with idiopathic sudden sensorineural hearing loss (ISSHL) was described in 14 included studies (Table 3). These studies reported increased signal intensity within the affected inner ear on 3D-FLAIR imaging taken before and after contrast administration. However, not all patients demonstrated increased signal intensity at either time point. Two studies noted that hyperintensity of the diseased ears was more prevalent in MRI acquired before contrast was administered, as opposed to images taken shortly after or at 4 h after GBCA injection (44, 54). Others reported an increased prevalence of abnormal enhancement of diseased ears when MRI was performed 4 h after GBCA administration as compared to MRI performed shortly after GBCA administration (58, 64). One study reported a correlation between increased signal in the diseased ear and poorer prognosis, but the association was only observed for pre-contrast imaging (44). Another study described a poorer prognosis when increased signal was observed on MRI performed 4 h after contrast administration (69). A third study reported a correlation between enhancement seen on MRI taken shortly after contrast injection and poorer initial hearing level but did not find a correlation with prognosis for hearing recovery (54).

**Table 3:** Summary of findings in studies of idiopathic sudden sensorineural hearing loss and vestibular neuritis.

**Idiopathic sudden sensorineural hearing loss and vestibular neuritis**								
**Author**	***n***	**Controls (*****n*****)**	**MRI Timing (time after contrast)**	**MRI protocol**	**Gd agent (dose in mmol/kg)**	**Signal assessment**	**Findings regarding enhancement**	**Correlation with symptoms and prognosis**
Stokroos et al. (37)	27	ND	Pre, Post (ND)	>1T MRI, T1W	ND	Qualitative	1 (3.7%) had high signal intensity pre- and post-contrast enhancement.	ND
Strupp et al. ^*^(38)	60	ND	Post (ND)	1.5T, T1W and T2W	Gd-DTPA (0.2)	Qualitative	No enhancement in any patient (0%).	ND
Cadoni et al. (41)	54	ND	Pre, Post (ND)	1.5T, T1W and 3D-FLAIR	Gd-DTPA (ND)	Qualitative	2 (3.7%) had pre-contrast high signal intensity, 1 (1.9%) had post-contrast enhancement.	ND
Sugiura et al. (42)	8	Contralateral ear (8)	Pre, Post (10 min)	3T, 3D-FLAIR	Gadodiamide (0.1)	Qualitative	4 (50%) had pre-contrast high signal intensity, 1(12.5%) had enhancement at 10 min.	2 (100%) patients with vertigo had pre-contrast high signal intensity. Patient with post-contrast enhancement had poor outcome.
Yoshida et al. (44)	48^**^	Contralateral ear (48)	Pre, Post (10 min)	3T, 3D-FLAIR	Gadodiamide (0.1)	Qualitative	31 (65%) had pre-contrast high signal intensity, 16 (33%) had enhancement at 10 min.	8 (80%) with high signal intensity in labyrinth had vertigo. High signal intensity pre-contrast, not post-contrast, correlated with worse prognosis.
Tagaya et al. (48)	10	Contralateral ear (9)	Pre, Post (4 h)	3T, 3D-FLAIR	Gadoteridol (0.1,0.2)	Quantitative	5/10 (50%) of patients had signal enhancement over controls after 4 h.	ND
Berrettini et al. (54)	23	Healthy controls and contralateral ear (20)	Pre, Post (ND)	3T, 3D-FLAIR	Gadobutrol (0.1)	Qualitative	13 (57%) had pre-contrast high signal intensity, 8 (35%) had post-contrast enhancement.	Patients with pre-contrast high signal intensity had lower initial hearing levels. Enhancement pattern not correlated with prognosis.
Kim et al. (56)	30	Contralateral ear (30)	Post (10 min, 4 hr)	3T, 3D-FLAIR	Gd-DTPA (0.2)	Quantitative	Enhancement in affected ears was only greater than unaffected at 10 min.	ND
Tanigawa et al. (57)	11 pre, 18 post	ND	Pre, Post (ND)	3T, 3D-FLAIR	Gadodiamide (0.1)	Qualitative	2 (11%) had pre-contrast high signal intensity, 1 (9%) had post-contrast enhancement.	High signal intensity only seen in patients with more severe impairment. Patient with post-contrast enhancement had significant improvement
Liao et al. (58)	54	Contralateral ear (54)	Pre, Post (10 min)	1.5T, 3D-FLAIR, 3D-FIESTA-C, FSPGR	Gadobutrol (0.1)	Quantitative, Qualitative	Visual: 32 (59%) had pre- and post-contrast high signal intensity. Quantitative: 43 (80%) had pre-contrast high signal intensity, 37 (69%) had post-contrast enhancement.	Degree of enhancement asymmetry correlated to final hearing loss.
Pakdaman et al. (61)	11	Contralateral ear (32)	Post (4 h)	3T, hT2W-3D-FLAIR	Gadopentetate dimeglumine (0.2)	Quantitative	No significant signal difference between affected and contralateral ears.	ND
Byun et al. ^*^(64)	29	Contralateral ear (29)	Pre, Post (10 min, 4 h)	3T, 3D-FLAIR	Gd-DTPA (0.2)	Qualitative	3 (10%) had enhancement at 10 min, 20 (69%) had enhancement at 4 h.	Duration of spontaneous nystagmus was correlated to enhancement at 4 h.
Eliezer et al. ^*^(68)	30	Healthy controls (26)	Post (4 h)	3T, 3D-FLAIR	Gadobutrol (0.1)	Qualitative	26 (87%) had post-contrast enhancement.	ND
Wang et al. (69)	100	Contralateral ear (100)	Post (4 h)	3T, 3D-FLAIR	Meglumine gadopentetate (0.2)	Quantitative, Qualitative	65 (65%) had post-contrast enhancement.	Enhancement correlated to more severe hearing loss. Degree of enhancement asymmetry correlated to final hearing loss.

*Studies designated with*

(*) *indicate studies of patients with vestibular neuritis. All other studies included in the table involved patients with idiopathic sudden sensorineural hearing loss (ISSHL). Data not included by authors in each study is depicted as “not described” (ND). Under the column MRI delay, “ND” is used to describe studies that did not report a specific delay time and is assumed to have performed MRI immediately after contrast injection. “Pre” indicates a scan was performed prior to the administration of contrast. Quantitative signal assessment methods involve the use of signal intensity measurements with regions of interest within the inner ear as compared to other imaged regions such as the cerebellum*.

** *eight patients from Suguira 2006. T, tesla; Gd, gadolinium; FLAIR, fluid-attenuated inversion recovery; FIESTA, fast imaging employing steady-state acquisition; hT2W, heavily T2-weighted; FSPGR, fast spoiled gradient-echo; Gd-DTPA, gadolinium with diethylenetriaminepentacetate; T1W, T1-weighted; T2W, T2-weighted*.

MRI findings in patients with Ménière's disease were described in 12 studies (Table 4). These studies reported increased enhancement of the affected ear in MRI acquired 4 h after contrast but no abnormal hyperintensity in imaging before contrast or at imaging 10 min after contrast. One study compared imaging taken 10 min and 4 h after contrast injection and reported an increase in signal intensity over controls only at 4 h (52). This observation was supported by one other study; however, this study only included one patient with unilateral definite Ménière's disease, and three with possible Ménière's disease (53). One study reported a correlation between enhancement on imaging 4 h after contrast and both the degree of hearing loss and severity of endolymphatic hydrops (73), while another study found no association between enhancement and hearing loss (50).

**Table 4:** Summary of findings in studies of Ménière's disease.

**Ménière's disease**								
**Author**	***n***	**Controls (*****n*****)**	**MRI delay**	**MRI protocol**	**Gd agent (dose)**	**Signal assessment**	**Findings regarding enhancement**	**Correlation with symptoms**
Fitzgerald et al. (36)	13	ND	Pre, Post (ND)	1.5T, T2W	ND	Qualitative	1 (8%) had abnormal MRI findings.	ND
Carfrae et al. (8)	7	Healthy controls (4)	Post (4 h)	3T, T1W	Gadodiamide (0.3)	Qualitative	All (100%) patients and controls had enhancement by 4 h.	ND
Suzuki et al. (50)	32	ND	Post (4 h)	hT2W-3D- FLAIR and 3D- FLAIR, 3T	Gadoteridol (0.1) and Gadodiamide (0.2)	Quantitative	Signal intensity was higher in patients who received a double dose vs. single dose of contrast.	No correlation between hearing level and signal intensity.
Tagaya et al. (48)	12	Contralateral ear (10)	Post (4 h)	3D FLAIR and 3D rIR, 3T	Gadoteridol (0.2)	Quantitative	Signal intensity of diseased ears was higher than contralateral ears.	ND
Sano et al. (53)	6	Contralateral ear (7)	Post (10 min, 4 h)	hT2W-3D- FLAIR	Gadodiamide (0.1)	Quantitative	Signal intensity of diseased ears greater than contralateral at 4 h but not 10 min in definite and possible Ménière's.	ND
Naganawa et al. (52)	10	ND	Pre, Post (10 min, 3.5–4.5 h)	3T, hT2W-3D-FLAIR	Gadodiamide (0.1)	Quantitative	No pre-contrast increased signal intensity or 10 m enhancement. Increased signal intensity seen at 3.5–4 h.	ND
Naganawa et al. (60)	9	Healthy controls (8)	Post (4 h)	hT2W-3D-FLAIR, 3T	Gadodiamide (0.1)	Quantitative	Signal intensity of disease ears not higher than controls.	ND
Pakdaman et al. (61)	32	Contralateral ear (43)	Post (4 h)	hT2W-3D-FLAIR, 3T	Gadopentetate dimeglumine (0.2)	Quantitative	Symptomatic ears had higher signal intensity than contralateral ears.	All ears with symptomatic hydrops had enhancement
Attye et al. (62)	200	Healthy controls (30)	Post (4.5–5.5 h)	3T, 3D-FLAIR	Gadoterate meglumine (0.1)	Qualitative	15 (7.5%) had enhancement of the semicircular canals.	ND
Eliezer et al. (65)	20	Contralateral ear (20)	Post (4 h)	3T, 3D-FLAIR	Gd-DOTA and Gadobutrol (0.1,0.2)	Quantitative, Qualitative	No difference in signal intensity between symptomatic and asymptomatic for either Gd agent (*p* = 0.14).	ND
Bernaerts et al. (66)	78	Contralateral ear (78)	Post (4 h)	3T, 3D-FLAIR	Gadobutrol (0.2)	Qualitative	51 (65%) symptomatic ears had enhancement, 2 (2.6%) contralateral ears had enhancement.	ND
Kahn et al. (73)	31	Healthy controls^***^ (23)	Post (4 h)	3T, 3D-FLAIR	Gadobutrol (0.1)	Qualitative	26/35 (74%) symptomatic ears had enhancement, 2/27 (7.4%) asymptomatic ears of Meniere's patients had enhancement. No (0%) enhancement in control ears.	Enhancement correlated to hearing level but not duration of disease

*Data not included by authors in each study is depicted as “not described” (ND). Under the column MRI delay, “ND” is used to describe studies that did not report a specific delay time and is assumed to have performed MRI immediately after contrast injection. “Pre” indicates a scan was performed prior to the administration of contrast. Quantitative signal assessment methods involve the use of signal intensity measurements with regions of interest within the inner ear as compared to other imaged regions such as the cerebellum*.

*** *Control ears were asymptomatic ears of patients with unilateral disease (hearing loss, vestibular neuritis), T, tesla; Gd, gadolinium; FLAIR, fluid-attenuated inversion recovery; FIESTA, fast imaging employing steady-state acquisition; hT2W, heavily T2-weighted; FSPGR, fast spoiled gradient-echo; Gd-DTPA, gadolinium with diethylenetriaminepentacetate; DOTA, dodecane tetraacetic acid; rIR, real inversion recovery; T1W, T1-weighted; T2W, T2-weighted*.

Five included studies examined MRI findings in patients with vestibular schwannoma (Table 5). Two studies reported enhancement of inner ear structures 7–10 min after contrast administration (45, 46). This was supported by another study which found decreased signal on 3D-FIESTA shortly after contrast injection which, similar to hyperintensity on 3D-FLAIR, suggests increased protein content in the inner ear (55). Another study reported a greater enhancement 5–8 h after contrast injection as compared to imaging immediately after contrast injection (70). Signal intensity at 5–8 h was correlated with poorer hearing as measured by pure tone thresholds and word recognition scores (70). The other studies with imaging performed shortly after GBCA administration reported no correlation between signal intensity and hearing level or tumor size (45, 46).

**Table 5:** Summary of findings in studies of vestibular schwannoma.

**Vestibular schwannoma**								
**Author**	***n***	**Controls (n)**	**MRI delay**	**MRI protocol**	**Gd agent (dose)**	**Signal assessment**	**Findings regarding enhancement**	**Correlation with symptoms**
Yamazaki et al. (45)	28 pre, 18 post	Contralateral ear (28)	Pre, Post (10 min)	3D-FLAIR, 3D-T2W, 3 T/1.5 T	Gd-DTPA or Gadodiamide (0.1)	Quantitative	Pre- and post-contrast signal intensity of affected ears was higher than controls.	Pre- and post-contrast signal intensity not correlated to hearing level
Lee et al. (46)	34	Contralateral ear (34)	Post (7 min)	3T, 3D-FLAIR	Gadopentetate dimeglumine (0.1)	Quantitative, Qualitative	Visual: 33 (97%) had cochlear enhancement, 31 (94%) had vestibular enhancement. Quantitative: Signal intensity was higher in affected ears.	No correlation between signal intensity and degree of hearing loss
Ishikawa et al. (55)	21	Normal controls (27)	Post (ND)	3D-FIESTA	Gd-DTPA or Gadodiamide (0.1)	Quantitative, Qualitative	Visual: 20 (95%) had decreased signal compared to controls. Quantitative: Affected ears had decreased signal intensity.	ND
Bowen et al. (70)	8	ND	Pre, Post (10 min, 5–8 h)	3T, 3D-FLAIR	ND	Quantitative	2 (25%) had enhancement at 10 min, 6 (75%) at 5–8 h. Signal intensity at 5–8 h was higher than at 10 min.	Signal intensity at 5–8 h correlated to word recognition scores but not initial symptoms, tumor size, or tumor growth.

*Data not included by authors in each study is depicted as “not described” (ND). Contrast delay described as “ND” is used to describe studies that did not report a specific delay time and is assumed to have performed MRI immediately after contrast injection. Quantitative signal assessment methods involve the use of signal intensity measurements with regions of interest within the inner ear as compared to other imaged regions such as the cerebellum. T, tesla; Gd, gadolinium; FLAIR, fluid-attenuated inversion recovery; FIESTA, fast imaging employing steady-state acquisition; hT2W, heavily T2-weighted; FSPGR, fast spoiled gradient-echo; Gd-DTPA, gadolinium with diethylenetriaminepentacetate; T2W, T2-weighted*.

Four studies reported on MRI findings in patients with otosclerosis (Table 6). Two described increased signal intensity on MRI before and shortly after contrast administration (59, 63). Two studies reported a correlation between enhancement and disease stage (60, 63), while another study reported no correlation between enhancement and degree of hearing loss (74).

**Table 6:** Summary of findings in studies of otosclerosis.

**Otosclerosis**								
**Author**	***n***	**Controls (*****n*****)**	**MRI delay**	**MRI protocol**	**Gd agent (dose)**	**Signal assessment**	**Findings regarding enhancement**	**Correlation with symptoms**
Lombardo et al. (59)	11	Matched controls (11)	Pre, post (ND)	3D-FLAIR, 3T	Gadoterate meglumine (0.1)	Quantitative, Qualitative	9 (82%) had pre-contrast enhancement, 8 (73%) had post-contrast enhancement.	ND
Berrettini et al. (63)	38	Healthy controls (11)	Pre, Post (ND)	3T, 3D-FLAIR	Gadoterate meglumine (0.1)	Quantitative, Qualitative	26 (68%) had pre-contrast enhancement, 14 (37%) had post-contrast enhancement.	Post-contrast enhancement correlated to more advanced disease.
Naganawa et al. (60)	12	Healthy controls (8)	Post (4 h)	hT2W-3D-FLAIR, 3T	Gadodiamide (0.1)	Quantitative	Signal intensity of diseased ears was higher than controls.	Signal intensity correlated to more advanced disease.
Laine et al. (74)	29	Healthy controls^***^	Post (4 h)	3T, 3D-FLAIR	Gadobutrol (0.1)	Quantitative, Qualitative	8 (21%) of affected ears had visual enhancement. Signal intensity of affected ears was higher than contralateral ears.	No correlation between signal intensity and level of hearing loss or vertigo.

*Data not included by authors in each study is depicted as “not described” (ND). Contrast delay described as “ND” is used to describe studies that did not report a specific delay time and is assumed to have performed MRI immediately after contrast injection. Quantitative signal assessment methods involve the use of signal intensity measurements with regions of interest within the inner ear as compared to other imaged regions such as the cerebellum*.

*** *Control ears were asymptomatic ears of patients with unilateral disease (acute vestibular syndrome). T, tesla; Gd, gadolinium; FLAIR, fluid-attenuated inversion recovery; hT2W, heavily T2-weighted*.

Our review also included 10 studies reporting abnormal inner ear enhancement in patients with sudden facial nerve paralysis (34, 35, 47), DFNA9 (mutation in the COCH gene) (67), Cogan syndrome (25), viral and bacterial labyrinthitis (23, 24, 30), and other inner ear abnormalities (39, 43, 49, 72).

## Discussion

Damage to the blood-labyrinth barrier has been implicated in the pathophysiology of inner ear disorders such as Ménière's disease and ISSHL (54, 75). Recently, investigators have begun using intravenous contrast-enhanced MRI to assess the permeability of this barrier as a potential tool in research and for diagnosis. The goal of this review was to evaluate current evidence for the role of gadolinium-based contrast in understanding blood-labyrinth barrier function and its utility in evaluating the integrity of this barrier in disease states of the inner ear.

Studies of both healthy and diseased ears of animals and humans demonstrated enhancement of inner ear structures following the administration of IV-gadolinium. In healthy ears of mice and guinea pigs, enhancement peaked and plateaued at 100 min after contrast administration. Further studies in animal ears affected by mechanical trauma (14) and inflammation (22) demonstrated increased enhancement, suggesting that disease states may increase the inner ear's permeability to gadolinium-based agents, potentially through alterations in the blood-labyrinth barrier. Studies have not sought to identify the mechanism(s) by which gadolinium crosses the blood-labyrinth barrier. Notably, these animal studies used an MRI magnetic field strength of at least 4.7 T, which exceeds the standard used in most human studies (1.5 and 3.0 T). Animals were also administered gadolinium at doses that were up to 15 times greater than the clinical standard of 0.1 mmol/kg for humans. Studies in healthy humans reported reliable gadolinium uptake within the inner ear 4 h after injection with either 0.1 (33) or 0.3 (8) mmol/kg gadolinium using a 3 T MRI scanner.

Pathologies such as ISSHL, Ménière's disease, otosclerosis, and vestibular schwannoma have been shown to alter this baseline GBCA enhancement by increasing the intensity of signal on MRI within inner ear structures. Although abnormal enhancement was well-described by most studies, there were conflicting reports of correlations between this enhancement and clinical characteristics such as prognosis and disease severity.

Although increased enhancement was common in these diseases, there were distinct differences by disease in the time of the observed increased signal. In both ISSHL and otosclerosis, hyperintense inner ear signal was present even before contrast was administered and just minutes after GBCA injection. Meanwhile, enhancement in patients with Ménière's disease or vestibular schwannoma was reported after a delay of four or more hours from contrast administration. Hyperintensity prior to contrast injection in cerebrospinal fluid has been hypothesized to result from increased protein content and could be a hypothesis for the increased pre-contrast signal seen in some patients with ISSHL and otosclerosis (76). Quicker uptake of contrast, or increased baseline signal, could indicate more severe blood-labyrinth barrier damage resulting in greater passage of GBCA and possibly protein into the perilymph. Alternatively, GBCA could have different routes of entry into perilymph, depending on the pathophysiology of the disease. The kinetics of enhancement with gadolinium could be important for evaluating the blood-labyrinth barrier in different disease states. However, due to the current paucity of studies on the time course of enhancement in disease, it is impossible to make a reliable comparison of blood-labyrinth barrier permeability in different disease states. Only two studies with few patients reported on enhancement findings at two time points in patients with Ménière's disease (52). While this early data is promising, studies with images taken at multiple time points are needed to better describe differences in the pattern of enhancement among different inner ear diseases compared to control ears.

Our analysis of the evidence was hampered by study design. Most notably there was a lack of adequate control groups, sample size justifications, and clear patient recruitment details. Compounding these quality issues was the substantial degree of variability in study protocols—particularly regarding the contrast agents used and the methods for determining enhancement. Counter et al. demonstrated that although linear and macrocyclic GBCA agents were useful in enhancing the inner ear structures, there was a distinct heterogenicity in kinetics (20). This makes it difficult to compare studies using different GBCA agents, given the importance of consistent enhancement kinetics when comparing uptake patterns in different disease states.

Additionally, there was no consensus on how to distinguish between normal and abnormal enhancement patterns. In studies that used controls, either from contralateral ears or healthy volunteers, some studies used subjective, visual assessment. Others used more objective measures of signal intensity normalized to signal from regions of the scan that were presumably unaffected by the disease. The consistency of control groups used in studies on systemic diseases or disease that can affect both ears such as Ménière's disease is particularly important. One included study found that asymptomatic ears of patients with Ménière's disease had increased signal intensity on contrast-enhanced MRI compared to controls (61), while another study described contrast enhancement of the asymptomatic ear in two patients with clinically unilateral Ménière's disease (66).

The goal of this scoping review was to evaluate the usefulness of IV-gadolinium MRI in assessing blood-labyrinth barrier permeability in healthy and diseased ears. Following intravenous administration of GBCA, GBCA enters the inner ear in healthy ears after a delay, presumably through the blood-labyrinth barrier. Despite these observations in healthy animals and humans, we found no conclusive evidence to support the assumption that gadolinium can be used to directly measure the health of the blood-labyrinth barrier. Our review also found a lack of studies that could correlate abnormal enhancement of the inner ear with a more general breakdown of the blood labyrinth barrier. While there is compelling evidence demonstrating increased GBCA uptake in diseased ears, future work must be done to clarify if any connection exists between abnormal enhancement and breakdown of the blood-labyrinth barrier. Furthermore, different disease states may affect the blood-labyrinth barrier by different mechanisms. There is a gap in the current literature regarding the mechanisms of GBCA uptake into the perilymph, the time course of uptake in diseased human ears, and the natural course of GBCA efflux from the perilymph. Rigorous future studies with adequate controls, clear patient recruitment methods, and objective measures of enhancement patterns are needed to determine the utility of contrast-enhanced MRI in assessing the integrity of the blood-labyrinth barrier.

## Data Availability Statement

The original contributions presented in the study are included in the article/Supplementary Material, further inquiries can be directed to the corresponding author.

## Author Contributions

BW and CS conceived of the concept of the article and the study design, applied the article quality assessment, and performed the initial draft of the manuscript. BW designed Figure 1. CS performed the initial search, designed Figure 2, and constructed the tables. All authors reviewed the included articles. All authors reviewed and approved the final submitted version of the manuscript.

## Conflict of Interest

The authors declare that the research was conducted in the absence of any commercial or financial relationships that could be construed as a potential conflict of interest.
