# Supplementary material for: MRI With Gadolinium as a Measure of Blood-Labyrinth Barrier Integrity in Patients With Inner Ear Symptoms: A Scoping Review
Source: Front Neurol. 2021 May 20;12:662264. doi: 10.3389/fneur.2021.662264 (PMC8173087; doi:10.3389/fneur.2021.662264)
Supplement: Supplementary file 1 [file Data_Sheet_1.docx]

Supplementary Material

Search Strategy 10-5-2020

PUBMED

((("Ear, Inner" [Mesh] OR "Ears, Inner"[tw] OR "Inner Ears"[tw] OR "Inner Ear"[tw] OR "Ear, Internal"[tw] OR "Ears, Internal"[tw] OR "Internal Ear"[tw] OR "Internal Ears"[tw] OR "Labyrinth"[tw] OR "Labyrinths"[tw] OR "Membranous Labyrinth"[tw] OR "Labyrinth, Membranous"[tw] OR "Labyrinths, Membranous"[tw] OR "Membranous Labyrinths"[tw] OR "Bony Labyrinth"[tw] OR "Bony Labyrinths"[tw] OR "Labyrinth, Bony"[tw] OR "Labyrinths, Bony"[tw] OR "Cochlea"[tw] OR "cochleae"[tw] OR "Cochleas"[tw] OR "cochlear"[tw] OR "Vestibule"[tw] OR "Vestibules"[tw] OR "vestibular"[tw] OR "Semicircular canal"[tw] OR "Semicircular canals"[tw] OR "Semicircular-canal"[tw] OR "Semicircular-canals"[tw]) AND ("Magnetic Resonance Imaging"[Mesh] OR "magnetic resonance imaging"[tw] OR "mri"[tw] OR "mris"[tw])) AND ("Permeability" OR "Permeable" OR "Leakiness" OR "Leaky" OR "Barrier" OR "Blood labyrinth" OR "Blood-labyrinth" OR "blood-labyrinthine" OR "blood labyrinthine" OR "Blood perilymph" OR "Blood-perilymph" OR "blood-endolymph" OR "blood endolymph" OR "fluid-blood" OR "fluid blood" OR "uptake")) AND ("gadolinium"[tw] OR "contrast"[tw] or "contrast-medium"[tw] or "postcontrast"[tw] or "post-contrast"[tw])

EMBASE

('permeability':ti,ab,kw OR 'permeable':ti,ab,kw OR 'leakiness':ti,ab,kw OR 'leaky':ti,ab,kw OR 'barrier':ti,ab,kw OR 'blood labyrinth':ti,ab,kw OR 'blood-labyrinth':ti,ab,kw OR 'blood-labyrinthine':ti,ab,kw OR 'blood labyrinthine':ti,ab,kw OR 'blood perilymph':ti,ab,kw OR 'blood-perilymph':ti,ab,kw OR 'blood-endolymph':ti,ab,kw OR 'blood endolymph':ti,ab,kw OR 'fluid-blood':ti,ab,kw OR 'fluid blood':ti,ab,kw OR 'uptake':ti,ab,kw) AND ('gadolinium':ti,ab,kw OR 'contrast':ti,ab,kw OR 'contrast-medium':ti,ab,kw OR 'postcontrast':ti,ab,kw OR 'post-contrast':ti,ab,kw) AND ('magnetic resonance imaging':ti,ab,kw OR 'mri':ti,ab,kw OR 'mris':ti,ab,kw OR 'nuclear magnetic resonance imaging'/exp) AND ('inner ear'/exp OR 'ears, inner':ti,ab,kw OR 'inner ears':ti,ab,kw OR 'inner ear':ti,ab,kw OR 'ear, internal':ti,ab,kw OR 'ears, internal':ti,ab,kw OR 'internal ear':ti,ab,kw OR 'internal ears':ti,ab,kw OR 'labyrinth':ti,ab,kw OR 'labyrinths':ti,ab,kw OR 'membranous labyrinth':ti,ab,kw OR 'labyrinth, membranous':ti,ab,kw OR 'labyrinths, membranous':ti,ab,kw OR 'membranous labyrinths':ti,ab,kw OR 'bony labyrinth':ti,ab,kw OR 'bony labyrinths':ti,ab,kw OR 'labyrinth, bony':ti,ab,kw OR 'labyrinths, bony':ti,ab,kw OR 'cochlea':ti,ab,kw OR 'cochleae':ti,ab,kw OR 'cochleas':ti,ab,kw OR 'cochlear':ti,ab,kw OR 'vestibule':ti,ab,kw OR vestibules:ti,ab,kw OR 'vestibular':ti,ab,kw OR 'semicircular canal':ti,ab,kw OR 'semicircular canals':ti,ab,kw OR 'semicircular-canal':ti,ab,kw OR 'semicircular-canals':ti,ab,kw)

CINAHL PLUS

(TI ( "Ears, Inner" OR "Inner Ears" OR "Inner Ear" OR "Ear, Internal" OR "Ears, Internal" OR "Internal Ear" OR "Internal Ears" OR "Labyrinth" OR "Labyrinths" OR "Membranous Labyrinth" OR "Labyrinth, Membranous" OR "Labyrinths, Membranous" OR "Membranous Labyrinths" OR "Bony Labyrinth" OR "Bony Labyrinths" OR "Labyrinth, Bony" OR "Labyrinths, Bony" OR "Cochlea" OR "cochleae" OR "Cochleas" OR "cochlear" OR "Vestibule" OR "Vestibules" OR "vestibular" OR "Semicircular canal" OR "Semicircular canals" OR "Semicircular-canal" OR "Semicircular-canals" ) OR AB ( "Ears, Inner" OR "Inner Ears" OR "Inner Ear" OR "Ear, Internal" OR "Ears, Internal" OR "Internal Ear" OR "Internal Ears" OR "Labyrinth" OR "Labyrinths" OR "Membranous Labyrinth" OR "Labyrinth, Membranous" OR "Labyrinths, Membranous" OR "Membranous Labyrinths" OR "Bony Labyrinth" OR "Bony Labyrinths" OR "Labyrinth, Bony" OR "Labyrinths, Bony" OR "Cochlea" OR "cochleae" OR "Cochleas" OR "cochlear" OR "Vestibule" OR "Vestibules" OR "vestibular" OR "Semicircular canal" OR "Semicircular canals" OR "Semicircular-canal" OR "Semicircular-canals" ) OR MH Ear, Inner) AND (TI ( "magnetic resonance imaging" OR "mri" OR "mris" ) OR AB ( "magnetic resonance imaging" OR "mri" OR "mris" ) OR MH magnetic resonance imaging) AND (TX ( "gadolinium" OR "contrast" or "contrast-medium" or "postcontrast" or "post-contrast" ) OR AB ( "gadolinium" OR "contrast" or "contrast-medium" or "postcontrast" or "post-contrast" )) AND (TX "Permeability" OR "Permeable" OR "Leakiness" OR "Leaky" OR "Barrier" OR "Blood labyrinth" OR "Blood-labyrinth" OR "blood-labyrinthine" OR "blood labyrinthine" OR "Blood perilymph" OR "Blood-perilymph" OR "blood-endolymph" OR "blood endolymph" OR "fluid-blood" OR "fluid blood" OR “uptake”)
